# Intellectual Developmental Disorder of Autosomal Dominant 61 Caused by a MED13 Variant Presenting With Congenital Unilateral Sensorineural Hearing Loss: A Case Report

**DOI:** 10.7759/cureus.99683

**Published:** 2025-12-20

**Authors:** Kathleen Pinto Alberto, Sara S Goulart, Filipa Valentim, Nídia N Faria, André Amaral

**Affiliations:** 1 Family Medicine, Horta's Health Center, Faial Island Health Unit, Horta, PRT; 2 Family Medicine, Arrifes Health Center, São Miguel Island Health Unit, Ponta Delgada, PRT

**Keywords:** cochlear hypoplasia, cochlear nerve hypoplasia, frameshift variant, global developmental delay, intellectual developmental disorder, med13, mrd61, supernumerary maxillary central incisor, unilateral sensorineural hearing loss, whole-exome sequencing

## Abstract

We report the case of an eight-year-old male with global developmental delay (GDD), an intellectual developmental disorder (IDD), mild dysmorphic features, congenital unilateral sensorineural hearing loss (SNHL), and a supernumerary left maxillary central incisor. Whole-exome sequencing (WES), in a singleton analysis, identified a heterozygous frameshift variant in MED13 (RefSeq NM_005121.2:c.2691del, p.(Asp898IlefsTer14)), confirmed de novo by parental testing and classified as pathogenic. Pathogenic MED13 variants can cause the rare neurodevelopmental disorder IDD, autosomal dominant 61 (MRD61; OMIM 618009), also referred to as MED13-related syndrome. The patient’s phenotype overlapped with those of previous cases but also included congenital unilateral SNHL and a supernumerary left maxillary central incisor. These findings may expand the known phenotypic spectrum of MRD61. This case is also consistent with haploinsufficiency as the disease mechanism for truncating MED13 variants in MRD61, underscoring the importance of exome sequencing in patients with neurodevelopmental disorders and congenital anomalies.

## Introduction

Intellectual developmental disorder (IDD), autosomal dominant 61 (MRD61; OMIM 618009), also referred to as MED13-related syndrome, is a rare autosomal dominant disorder [1] caused by heterozygous pathogenic variants in the MED13 gene, which encodes a subunit of the Mediator complex that regulates transcription by RNA polymerase II [2]. This condition is rare in the peer-reviewed literature [1-10], and most reported variants are de novo [2], including both missense [2-7] and truncating [1,2,8] variants. The phenotype is heterogeneous and typically includes global developmental delay (GDD), IDD, mild dysmorphic features, and congenital anomalies [1-10]. Hearing involvement has been reported in MED13-related syndrome, including conductive hearing loss [2,3,9] and labyrinthine or ossicular malformations [9], whereas sensorineural hearing loss (SNHL) has been reported in MED13L-related syndrome [11]. It is important to document new cases of MED13-related syndrome, and here we describe a case of a child with GDD, IDD, mild dysmorphic features, congenital unilateral SNHL, and a supernumerary left maxillary central incisor, with an identified pathogenic MED13 variant. To the best of our knowledge, this is the first documented case of congenital unilateral SNHL and a supernumerary left maxillary central incisor in an individual with MRD61. These findings may further expand the phenotypic spectrum of MED13-related syndrome.

## Case presentation

The patient is an eight-year-old male, second-born to non-consanguineous Portuguese parents, born on Faial Island, Azores, Portugal. The pregnancy was uncomplicated, and delivery occurred at 39 weeks’ gestation via spontaneous vaginal delivery. The intrapartum and neonatal courses were uncomplicated. The birth anthropometrics were appropriate for gestational age. The patient was exclusively breastfed. The family history was noncontributory.

He was under general pediatric follow-up for congenital muscular torticollis, detected at birth. Beginning at approximately six to seven months of age, he developed repetitive head banging that disrupted sleep, with nocturnal awakenings, prompting referral to the neurodevelopmental pediatrics service for further evaluation and ongoing follow-up. By 18 months of age, caregivers noted episodes of excessive crying and laughter with no apparent motive that could precipitate this behavior. At the age of three, sleep initiation remained prolonged and was accompanied by poor sleep routines. The use of melatonin and tryptophan helped improve overall sleep. At the same age, his play interests were circumscribed, focusing on cars and rolling random objects. Low frustration tolerance was evident, with aggressive outbursts, including kicks, pinches, and hair pulling, when he was contradicted.

At three years of age, GDD was confirmed based on a multidisciplinary clinical assessment and standardized developmental evaluation, with performance equivalent to that of a 24-month-old child. He had marked locomotor limitations, including the inability to run or jump, difficulty kicking a ball, and impaired balance. He also had language limitations, producing fewer than eight intelligible words, and had limited naming and identification of common objects. He had no sphincter control and an immature capacity to feed and dress by himself, although he obeyed simple commands and engaged in basic interactive play. Tasks assessing concept formation, sequencing, early numeracy, and applied problem-solving were areas in which he was severely limited compared with what was expected at that age. He had good fine motor and visuomotor coordination, although his bimanual coordination was limited.

At the age of five years, he was diagnosed with mild IDD by a neurodevelopmental pediatrics service, based on clinical and standardized cognitive assessments. At the age of seven, he exhibited traits suggestive of autism spectrum disorder (ASD) or attention deficit hyperactivity disorder (ADHD), but a formal diagnostic evaluation is still ongoing. He achieved sphincter control, although intermittent nocturnal enuresis continued.

The craniofacial examination revealed preauricular pits, epicanthal folds, and short palpebral fissures bilaterally. The nasal morphology showed a broad tip and a short columella. His dentition was notable for a supernumerary left maxillary central incisor. Additional skeletal findings included ligamentous laxity of the small joints of the hands and bilateral clinodactyly of the fourth and fifth fingers.

**Figure 1:**
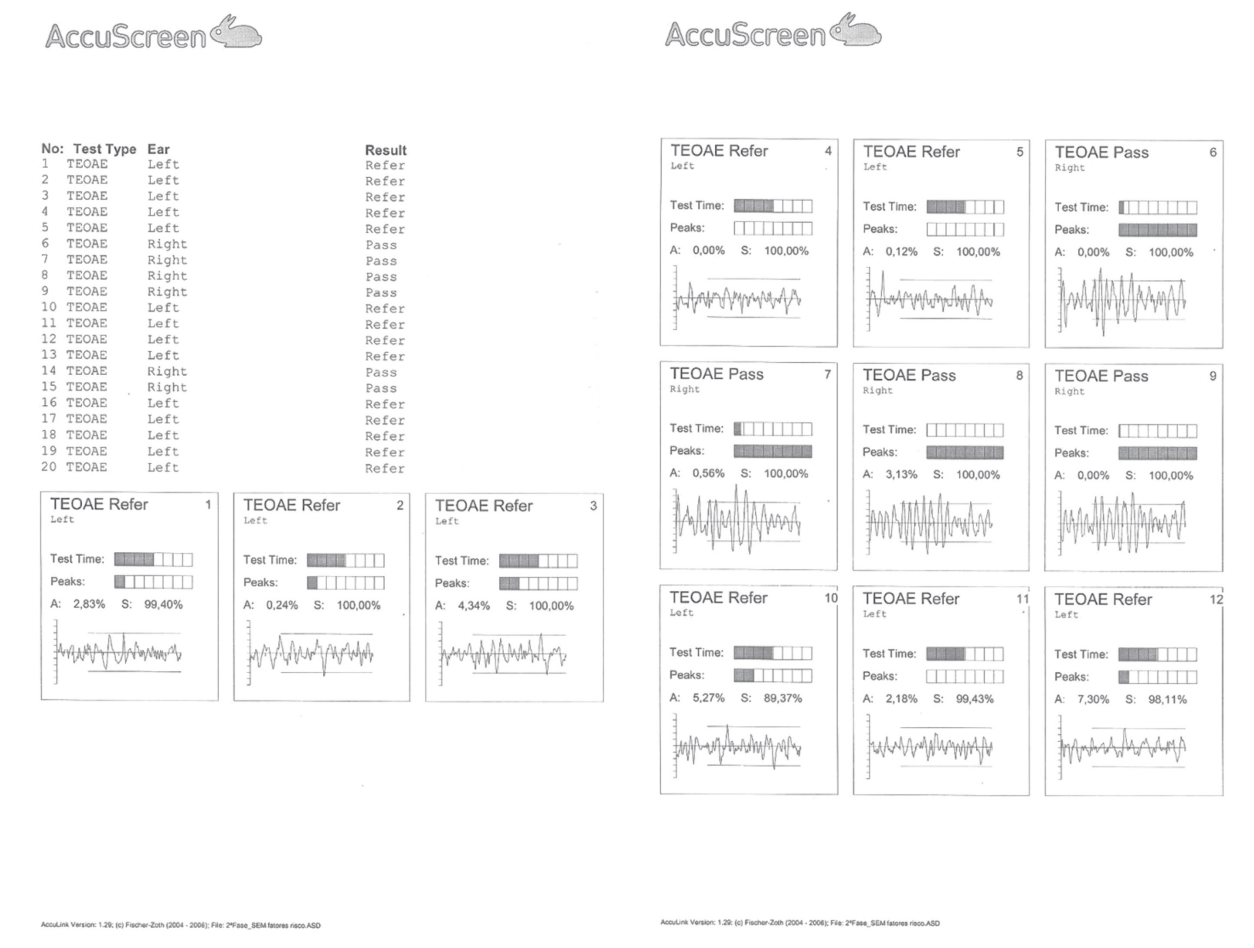
Newborn TEOAE hearing screening results. The summary table on the left lists 20 TEOAE screening tests, with the corresponding ear and outcome. The panels on the right display individual TEOAE test screens for the left and right ears, each indicating the ear tested, the overall automated result (“Pass” or “Refer”), test time, and the recorded response waveform. In the data displayed, all right-ear screenings are classified as “Pass.” In contrast, all left-ear screenings are classified as “Refer,” indicating repeated failure of the left ear on newborn hearing screening. TEOAE: transient-evoked otoacoustic emission; Pass: automated screening result indicating that the ear passed the screening; Refer: automated screening result indicating that the ear did not pass the screening.

Newborn hearing screening using transient-evoked otoacoustic emissions (TEOAEs) at one, two, and four months of age repeatedly failed in his left ear, whereas the right ear consistently passed (Figure 1).

**Figure 2:**
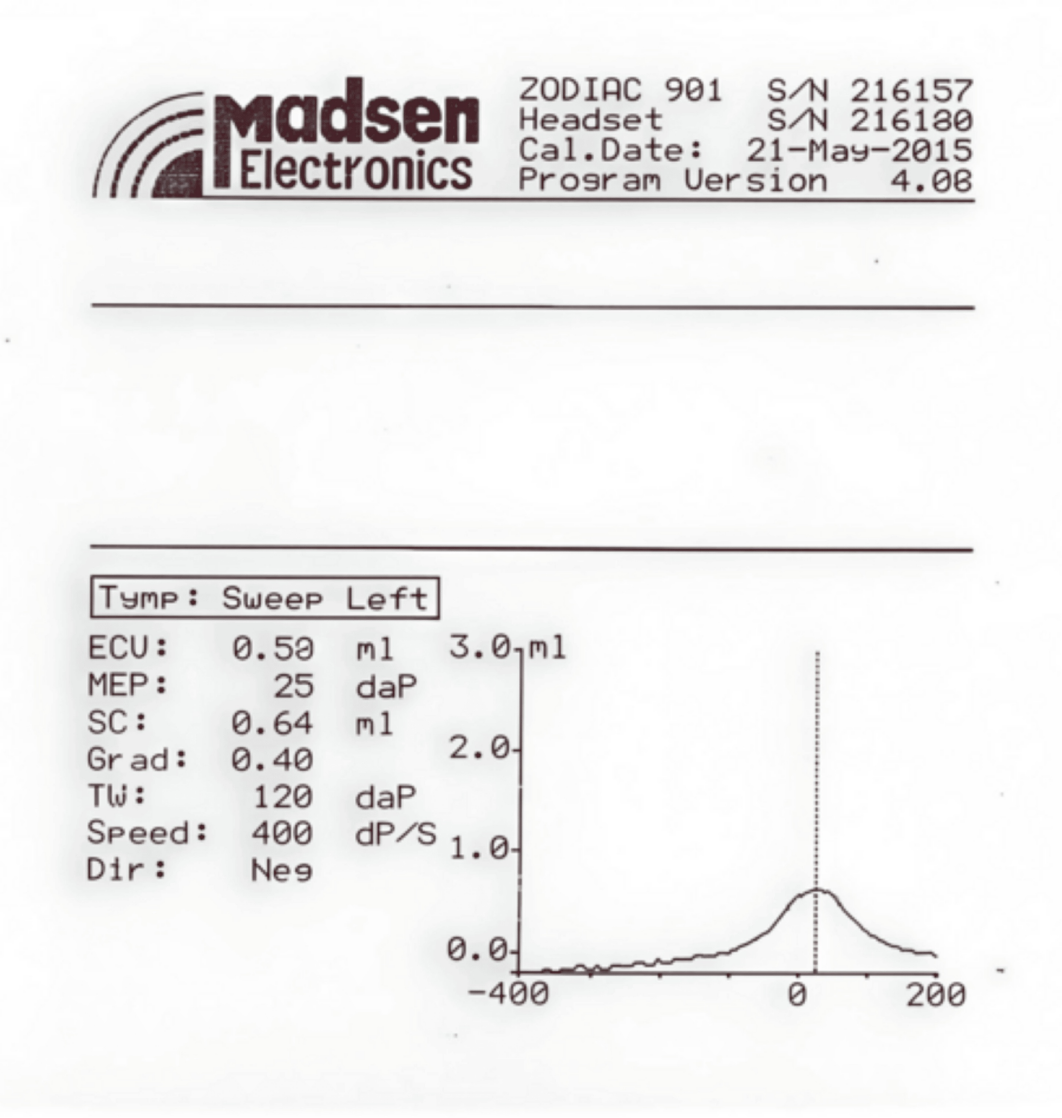
Tympanogram of the left ear at five months of age. The x-axis represents ear-canal air pressure in daPa (range -400 to +200), and the y-axis represents acoustic admittance in mL. The tracing shows a single-peaked tympanometric curve, with a peak near atmospheric pressure, consistent with a Type A tympanogram. Tympanometric parameters indicate an ECU of 0.50 mL, MEP of +25 daPa, SC of 0.64 mL, Grad of 0.40, and TW of 120 daPa. This pattern is consistent with normal middle-ear function, with no evidence of middle-ear effusion or of significant negative middle-ear pressure. ECU: equivalent ear canal volume; MEP: middle-ear pressure; SC: static compliance; Grad: tympanometric gradient; TW: tympanometric width; Speed: sweep pump speed (400 daPa/s); Dir: pressure sweep direction (Neg = negative direction); daPa: decapascals; mL: milliliters.

His repeated failed screenings in the left ear warranted a diagnostic audiologic evaluation to determine the presence of hearing loss. At five months of age, tympanometry of the left ear was performed, and the tracing demonstrated a Type A tympanogram, consistent with normal middle ear function (Figure 2).

**Figure 3:**
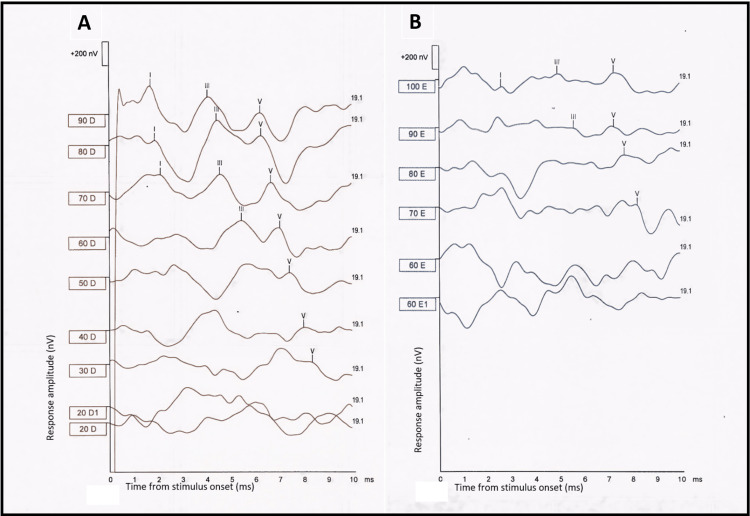
ABR at nine months of age. The x-axis represents time from stimulus onset, in ms, and the y-axis represents response amplitude, in nV. The vertical calibration bar corresponds to 200 nV. The value 19.1, printed next to each trace, indicates a stimulus repetition rate of 19.1 presentations per second. A) Multiple traces are displayed in a stacked format for stimulus intensities from 90 to 20 dB nHL, in 10-dB steps (labels: 90 D, 80 D, 70 D, 60 D, 50 D, 40 D, 30 D, 20 D1, and 20 D), with two recordings obtained at 20 dB nHL (20 D1 and 20 D). The numbers next to each trace indicate the stimulus level in dB nHL, and the letter D indicates the right ear. Waves I, III, and V are identifiable at higher intensities, and wave V remains identifiable down to 30 dB nHL, but is not evident in either trace at 20 dB nHL, providing an estimated electrophysiologic threshold of approximately 30 dB nHL. B) Multiple traces are displayed in a stacked format for stimulus intensities from 100 to 60 dB nHL, in 10-dB steps (labels: 100 E, 90 E, 80 E, 70 E, 60 E, and 60 E1), with two recordings obtained at 60 dB nHL (60 E and 60 E1). The numbers next to each trace indicate the stimulus level in dB nHL, and the letter E indicates the left ear. The waveforms show relatively poor morphology, with waves I, III, and V identifiable only at higher intensities. Wave V is evident at 100 to 70 dB nHL, but is not in either recording at 60 dB nHL, providing an estimated electrophysiologic threshold of approximately 70 dB nHL. ABR: auditory brainstem response; dB nHL: decibels normal hearing level; D: right ear, from the Portuguese word “direito”; ms: milliseconds; nV: nanovolts; E: left ear, from the Portuguese word “esquerdo”.

At nine months of age, an auditory brainstem response (ABR) test was performed during natural sleep, for diagnostic evaluation and to estimate ear-specific electrophysiologic thresholds. In the right ear, ABR waveforms were well-formed, yielding an estimated electrophysiologic threshold of 30 decibels normal hearing level (dB nHL), consistent with ABR parameters within normal limits in his right ear (Figure 3A). The left ear ABR waveforms were poorly formed, yielding an elevated electrophysiologic threshold of approximately 70 dB nHL (Figure 3B).

**Figure 4:**
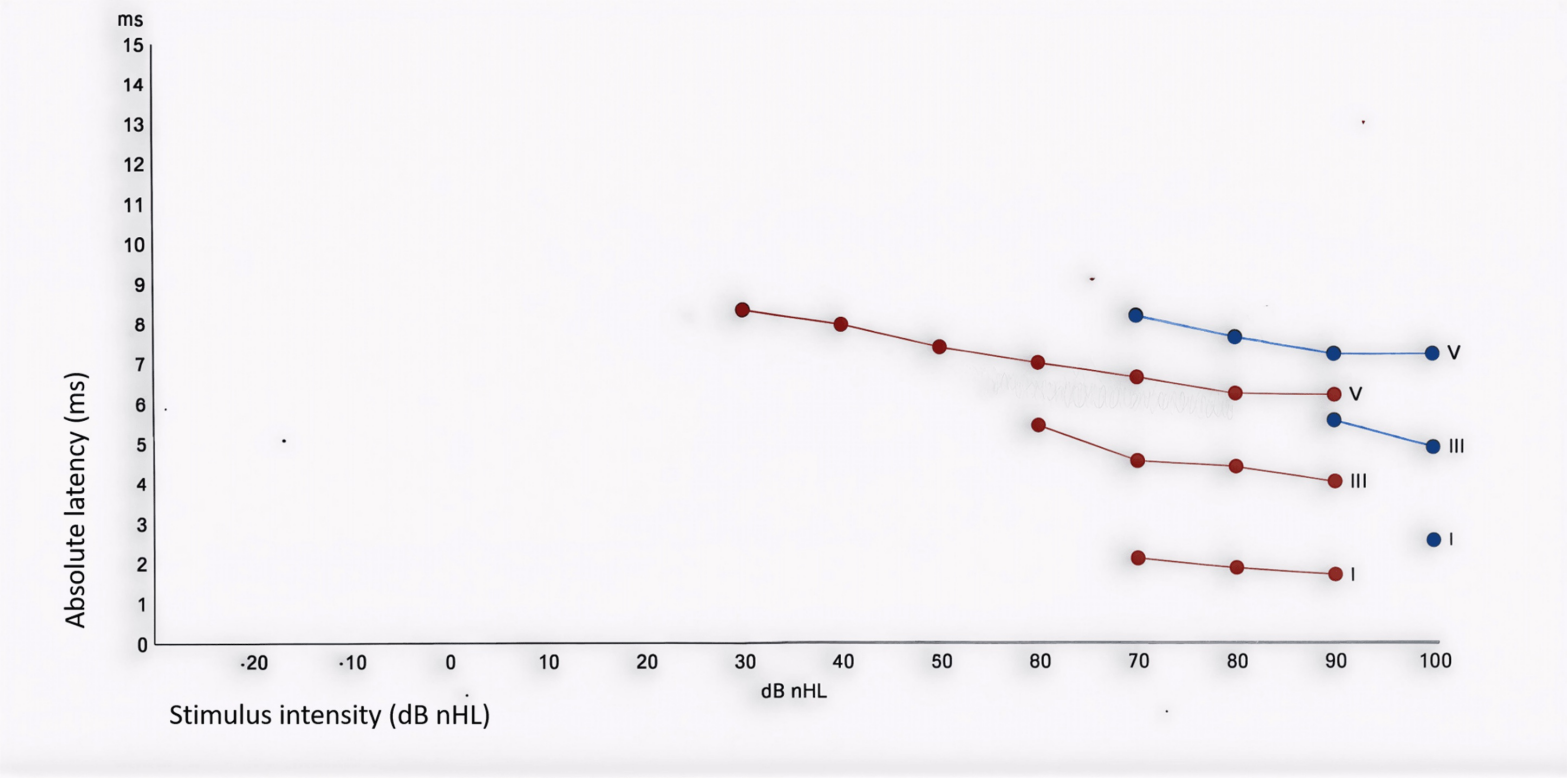
ABR latency-intensity functions of both ears at nine months of age. Latency-intensity functions for ABR waves I, III, and V in the right and left ears. Red dots and lines represent the right ear, and blue dots and lines represent the left ear. The x-axis represents stimulus intensity, in dB nHL, and the y-axis represents absolute wave latency, in ms. Data points plot the absolute latencies of waves I, III, and V at each recorded stimulus level, with lines connecting dots for the same wave and ear. The right ear shows the expected decrease in wave latency with increasing stimulus intensity. In contrast, left-ear latencies are systematically prolonged, and waves I, III, and V are only identifiable at higher stimulus levels, compared with the right ear. ABR: auditory brainstem response; dB nHL: decibels normal hearing level; ms: milliseconds.

The ABR latency-intensity function illustrated the inverse relationship between stimulus intensity, measured in dB nHL, and the absolute latency, measured in milliseconds (ms), of waves I, III, and V. The resulting lines showed a decrease in wave latencies with increasing stimulus intensities (Figure 4). The right ear showed the expected reduction in wave latencies as the intensity increased, whereas the left ear demonstrated prolonged or absent responses at comparable intensities. 

A detailed tabulation of ABR absolute latencies, interpeak latencies, and interaural latency differences provided absolute latency values (ms) for waves I, III, and V, as well as interpeak latency intervals (I-III, III-V, I-V) across stimulus intensity levels (dB nHL) and interaural latency differences, expressed as the absolute right-left latency difference (|R-L|, ms), for the corresponding measures (Table 1).

**Table 1:** ABR absolute latencies, interpeak latencies, and interaural latency differences of both ears at nine months of age. The “Stimulus condition” column lists the stimulus intensity (30-100 dB nHL) and the ear tested (L or R), with two recordings obtained at 60 dB nHL (60 L1 and 60 L). Under “Absolute and interpeak latencies (ms),” the table presents absolute latencies for waves I, III, and V, and the interpeak latency intervals I-III, III-V, and I-V, expressed in ms, at each recorded stimulus level. The “Interaural latency differences (|R-L|, ms)” columns list absolute latency differences between the right and left ears for a given wave or interpeak latency interval, expressed in ms, where responses were obtained in both ears. In the right ear (R), the stimulus intensity was not measured at 100 dB nHL; therefore, the cells that correlate the interaural latency difference between ears at 100 dB nHL are empty. The other empty cells indicate that no reliable waveform was identified at that intensity in that ear, or that an interaural difference could not be calculated because a response was absent in one ear. In the right ear, waves I, III, and V were robustly identified at higher intensities, with interpeak latencies (I-III, III-V, I-V) within normal limits and wave V present down to 30 dB nHL, consistent with ABR parameters within normal limits in the right ear. In the left ear, waves I, III, and V were identified only at 100 dB nHL, with waves III and V persisting at 90 dB nHL and an isolated wave V at 80 and 70 dB nHL. All waves were absent at 60 dB nHL, consistent with an elevated electrophysiologic threshold. Interaural analysis demonstrated increased wave V interaural latency differences, indicating that wave V in the left ear occurred later than in the right ear at matched stimulus intensities. ABR: auditory brainstem response; dB nHL: decibels normal hearing level; R: right ear; L: left ear; ms: milliseconds; |R-L|: absolute interaural latency difference between the right and left ears.

Stimulus condition	Absolute and interpeak latencies (ms)						Interaural latency difference (ǀR-Lǀ, ms)			
	I	III	V	I-III	III-V	I-V	I-III	III-V	I-V	V-V
100 L	2.57	4.90	7.23	2.33	2.33	4.66	-	-	-	-
90 R	1.70	4.03	6.20	2.33	2.17	4.5	-	0.50	-	1.03
90 L	-	5.57	7.23	-	1.66	-	-	0.50	-	1.03
80 R	1.87	4.40	6.23	2.53	1.83	4.36	-	-	-	1.44
80 L	-	-	7.67	-	-	-	-	-	-	1.44
70 R	2.10	4.53	6.63	2.43	2.10	4.53	-	-	-	1.57
70 L	-	-	8.20	-	-	-	-	-	-	1.57
60 R	-	5.43	7.00	-	1.57	-	-	-	-	-
60 L	-	-	-	-	-	-	-	-	-	-
60 L1	-	-	-	-	-	-	-	-	-	-
50 R	-	-	7.40	-	-	-	-	-	-	-
40 R	-	-	7.97	-	-	-	-	-	-	-
30 R	-	-	8.33	-	-	-	-	-	-	-

**Figure 5:**
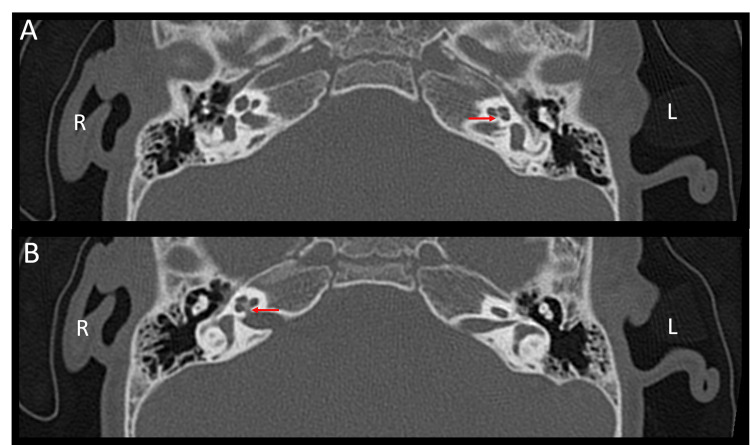
Axial CT images of the IAC. A) The left (L) bony cochlear nerve canal with apparent stenosis (red arrow). B) The right (R) bony cochlear nerve canal (red arrow) is visualized in the image. Image A was selected as the better plane for visualizing the left bony cochlear nerve canal, while image B was chosen to best demonstrate the right bony cochlear nerve canal, as both could not be adequately visualized in a single plane. CT: computed tomography; IAC: internal auditory canal; R: right side; L: left side.

**Figure 6:**
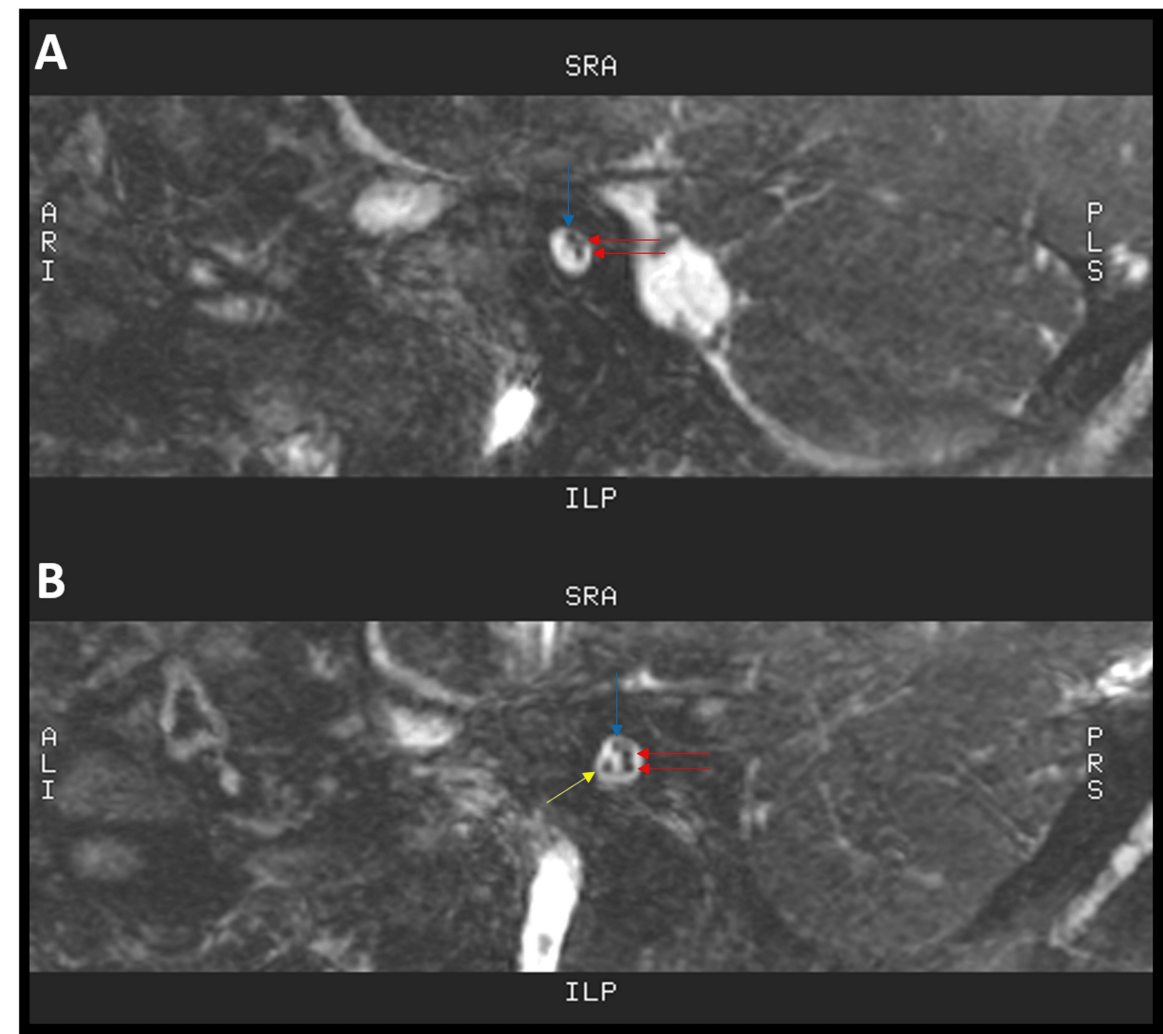
Sagittal oblique reformatted MRI images of the IAC. A) In this image, three nerves are visualized at the fundus of the left IAC: the facial nerve (blue arrow) superiorly, and the superior and inferior vestibular nerves (red arrows) posteriorly. The left cochlear nerve cannot be identified in this image. B) This image demonstrates the four nerves at the fundus of the right IAC: the facial nerve (blue arrow) superiorly, the cochlear nerve (yellow arrow) anteroinferiorly, and the superior and inferior vestibular nerves (red arrows) posteriorly. The letters shown on the MRI images (A, P, R, L, S, I) are orientation markers indicating anterior, posterior, right, left, superior, and inferior directions in the oblique sagittal plane. MRI: magnetic resonance imaging; IAC: internal auditory canal; A: anterior; P: posterior; R: right; L: left; S: superior; I: inferior.

**Figure 7:**
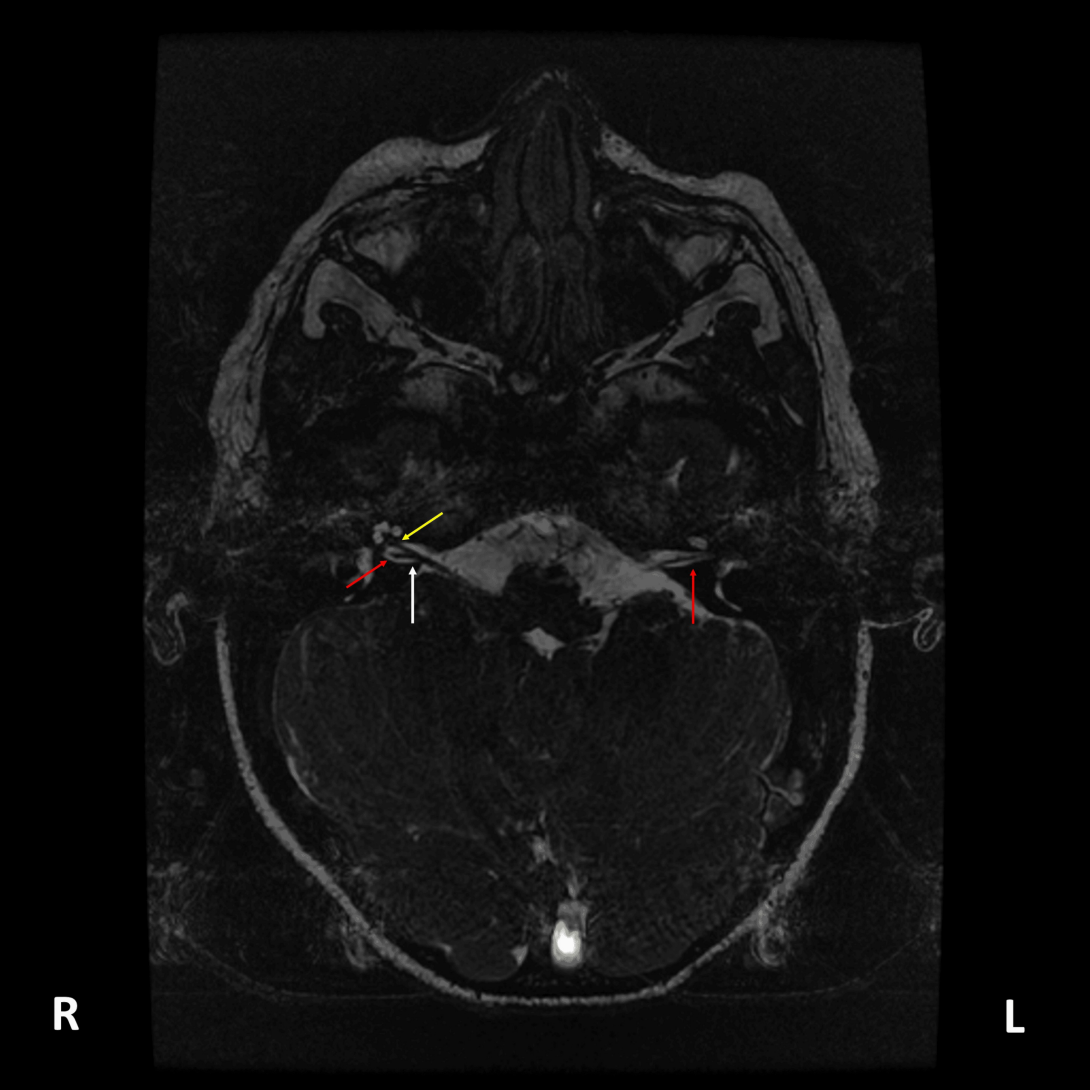
Axial FIESTA-C MRI image of the IAC. On the right (R) side, there is a clear separation of the vestibulocochlear nerve (white arrow) into the cochlear nerve anteriorly (yellow arrow) and the vestibular nerve posteriorly (red arrow). On the left (L) side, this separation of the vestibulocochlear nerve is not observed, and only the vestibular nerve is visualized (red arrow), showing apparent thinning compared with the contralateral side. FIESTA-C: fast imaging employing steady-state acquisition - C-variant; MRI: magnetic resonance imaging; IAC: internal auditory canal; R: right side; L: left side.

**Figure 8:**
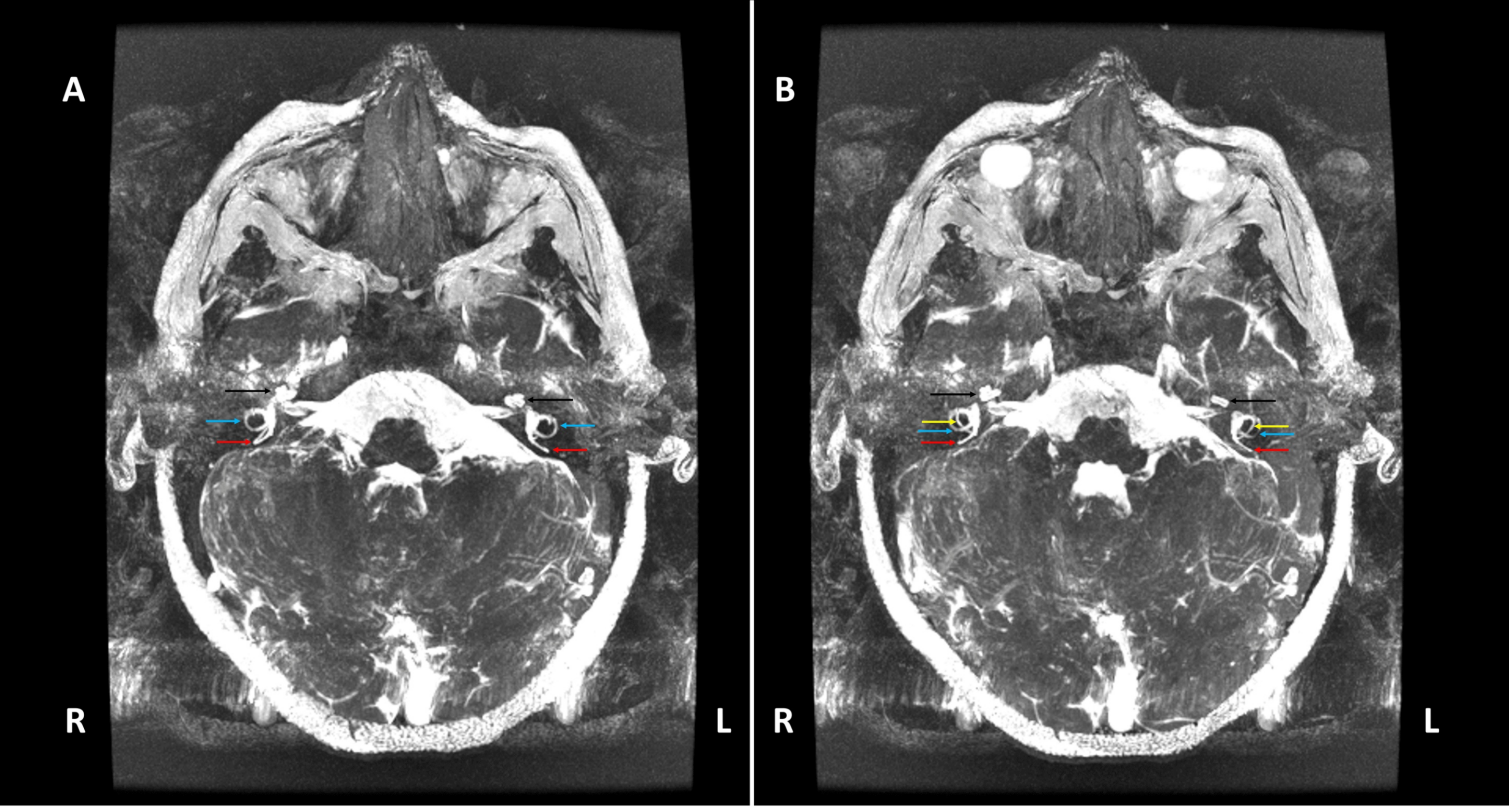
Axial MIP reconstruction of the FIESTA MRI images of the IAC. A) The image demonstrates preserved fluid signal, bilaterally, within the cochlear turns (black arrows), as well as within the posterior (red arrows) and lateral (blue arrows) semicircular canals. B) The subsequent image also demonstrates preserved fluid signal, bilaterally, outlining the posterior (red arrows), lateral (blue arrows), and anterior (yellow arrows) semicircular canals, as well as within the cochlear turns (black arrows). On the left (L) side, the cochlea appears smaller compared with the cochlea of the right (R) side. MIP: maximum intensity projection; FIESTA: fast imaging employing steady-state acquisition; MRI: magnetic resonance imaging; IAC: internal auditory canal; R: right side; L: left side.

Magnetic resonance imaging (MRI) of the brain and internal auditory canals (IACs), with adjunctive temporal bone computed tomography (CT), was performed when he was two years old. The report described a reduced caliber of the left IAC and stenosis of the left bony cochlear nerve canal (also known as the cochlear aperture) (Figure 5A), findings compatible with hypoplasia of the vestibulocochlear nerve (cranial nerve VIII), evidenced by a thin cisternal segment. The cochlear nerve was not visualized within the fundus of the left IAC (Figure 6A), and thinning of the ipsilateral vestibular nerve was also suspected (Figure 7). Additional left-sided findings included mild cochlear hypoplasia. Although the turns were formed, the middle and apical turns were smaller than usual (Figure 8). No significant abnormalities were observed on his right side (Figures 5B, 6B, 7, 8). The formal radiology report of the MRI and CT suggested hypoplasia of the left vestibulocochlear nerve, with apparent absence or marked hypoplasia of the cochlear nerve, and mild cochlear hypoplasia of the left inner ear.

His genetic diagnosis was established by whole-exome sequencing (WES), performed as a singleton analysis, and identified a heterozygous frameshift variant in MED13 (RefSeq NM_005121.2:c.2691del, p.(Asp898IlefsTer14)). This variant was classified as pathogenic. Previously, he had undergone genetic testing, including conventional karyotyping (46,XY), 180 K oligonucleotide array-CGH, an MLPA panel for deafness genes, and FMR1 CGG-repeat analysis, all of which were normal.

Due to the variable phenotypic expression of the MED13-related syndrome, he underwent additional evaluations, including transthoracic echocardiography, renal ultrasonography, and ophthalmological evaluation, all of which were normal. He is under the care of otolaryngology for chronic nasal obstruction, with hypoplastic frontal sinuses and marked nasal septal deviation, without significant nasal airway obstruction. He is currently undergoing follow-up with physical medicine and rehabilitation to monitor potential scoliosis. The patient received continuous multidisciplinary support. He is currently registered in a structured educational program and receives support for psychomotor therapy, speech therapy, and psychology.

## Discussion

The MED13 frameshift variant was classified according to the ACMG/AMP guidelines for variant interpretation [12]. The variant meets PVS1 (very strong) for a predicted loss-of-function allele in a gene for which haploinsufficiency is an established disease mechanism for MRD61, and PS2 (strong), based on confirmed de novo status (parentage confirmed, and both parents tested negative). Given the child's phenotypic concordance with the MED13-related syndrome, PP4 (supporting) was also applicable. Together, these criteria support the classification of this variant as pathogenic.

Repeated ‘Refer’ results for left ear TEOAEs raised suspicion of hearing loss or middle ear dysfunction, warranting further diagnostic evaluation. The tympanometry performed on his left ear demonstrated a Type A tympanogram, with no evidence of middle ear effusion or negative middle ear pressure. These findings were consistent with normal middle ear function and supported a sensorineural, rather than a conductive, etiology for unilateral hearing loss. The ABR was used to assess the integrity of neural conduction along the cochlear nerve and the ascending brainstem auditory pathways. In the left ear, the ABR waveforms showed poor morphology, with an estimated electrophysiologic threshold of approximately 70 dB nHL, consistent with significant SNHL. The normal TEOAEs, ABR, and imaging results in the right ear were consistent with the unilateral nature of the deficit. Although MRI and CT suggested absence or marked hypoplasia of the cochlear nerve, the ABR waveforms recorded from the left ear were compatible with hypoplasia, rather than aplasia, of the cochlear nerve. These audiologic findings in the left ear, together with imaging, are consistent with the diagnosis of congenital unilateral SNHL.

The patient’s phenotype matches previous descriptions of cases with variants in MED13 [1-10] but includes congenital unilateral SNHL and a supernumerary left maxillary central incisor, possibly expanding the phenotypic spectrum of MRD61.

To our knowledge, a standardized treatment approach for this disorder has not yet been established. Management is individualized and based on the patient’s phenotypic manifestations. He received ongoing multidisciplinary follow-up in neurodevelopmental pediatrics, otolaryngology, and physical medicine and rehabilitation.

This report adds to the evidence supporting haploinsufficiency as a likely pathogenic mechanism for truncating MED13 variants. It underscores the relevance of exome sequencing in the etiological diagnosis of unexplained congenital unilateral SNHL.

## Conclusions

This case of MED13-related syndrome, associated with a truncating MED13 variant, may further broaden the phenotypic spectrum by documenting congenital unilateral SNHL with mild left cochlear hypoplasia, left cochlear nerve hypoplasia, and a supernumerary left maxillary central incisor in the literature. Taken together with previous reports, these observations support haploinsufficiency as a likely disease mechanism for truncating mutations in MED13.

Given the rarity of MRD61, the description of this case contributes to the literature by documenting new alterations, and such data sharing is essential to increase knowledge and sensitize healthcare professionals to this type of genetic disease.
